# Cerebral hemorrhagic infarction as the initial manifestation of deep venous thrombosis in a child with patent foramen ovale

**DOI:** 10.21542/gcsp.2018.17

**Published:** 2018-06-30

**Authors:** Dimitrios Panagopoulos, Sofia Loukopoulou, Evagelos Karanasios, Georgia Grigoriadou, Nikolaos Eleftherakis

**Affiliations:** 1 Pediatric Hospital of Athens, “Aglaia Kyriakou”, Neurosurgical Department, Athens, Greece; 2 Cardiology Department, Pediatric hospital “Agia Sophia”, Athens, Greece; 3 Hemodynamic Cardiology Department, Pediatric hospital “Agia Sophia”, Athens, Greece

## Abstract

Arterial ischemic stroke (AIS), with an estimated incidence of 1.1–4.3 per 100,000, is an important cause of morbidity and mortality in children and the risk of recurrence is high. We present the case of an 11-year-old child who presented with a symptomatology of acute ischemic stroke of unknown etiology. The radiological investigation did not reveal any underlying brain abnormality that could cause the event. The diagnostic work up included an echocardiogram, which revealed a thrombus in the right atrium, in conjunction with a patent foramen ovale. The patient was initiated immediately on anticoagulation therapy with low molecular weight heparin and warfarin, but two days later she suffered pulmonary emboli, diagnosed with spiral thorax computed tomography (CT) scan. An ultrasound study of the vessels of the lower extremitiesgcsp201817-main-client.xml revealed deep venous thrombosis (DVT), which was considered to be the underlying causative mechanism.

## Introduction

To the best of our knowledge, this is the first documented case of right atrial thrombus resulting from deep venous thrombosis in a pediatric patient with patent foramen ovale and associated ischemic stroke event.

A discussion regarding the definition of cryptogenic stroke, its etiology and relationship with deep venous thrombosis and the currently proposed therapy, follows.

## Case report

We present a rare clinical case of a young girl harboring a latent deep venous thrombosis (DVT), a thrombus in the right atrium with subsequent arterial ischemic stroke (AIS) of the brain, possibly due to a patent foramen ovale (PFO). This was the first clinical manifestation of an otherwise unrecognized clinical condition.

**Figure 1.**
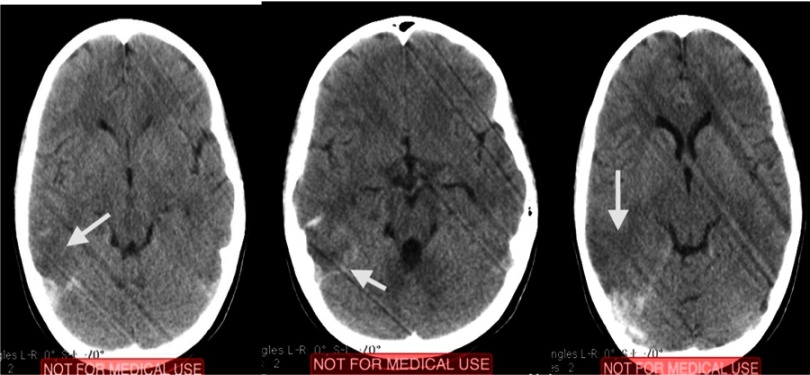
Initial CT scan, after the ictus. Arrows depict the areas of ischemic stroke (hypodense), with a surrounding area of hemorrhagic transformation.

An 11-year old girl presented with headache, vomiting, dizziness, dysphasia and gaze dedication for a few seconds. The initial computed tomography (CT) scan revealed intracerebral hematoma (ICH) in the right parieto-occipital region with perilesional edema (Figure 1).

**Figure 2.**
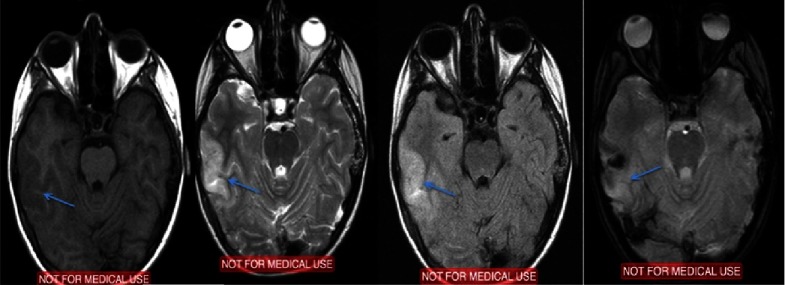
Initial MRI scan within one day after the event. Arrows depict the pathologic changes at T1W, T2W, FLAIR and T2 GRE sequences.

Patient was admitted in the neurosurgical clinic and was initiated on anticonvulsant medication. Neurological and ophthalmological examination did not reveal any focal deficits. MRI performed the same day revealed edematous configuration of the nearby gyri with concurrent presentation of hemorrhagic elements (Figure 2).

**Figure 3.**
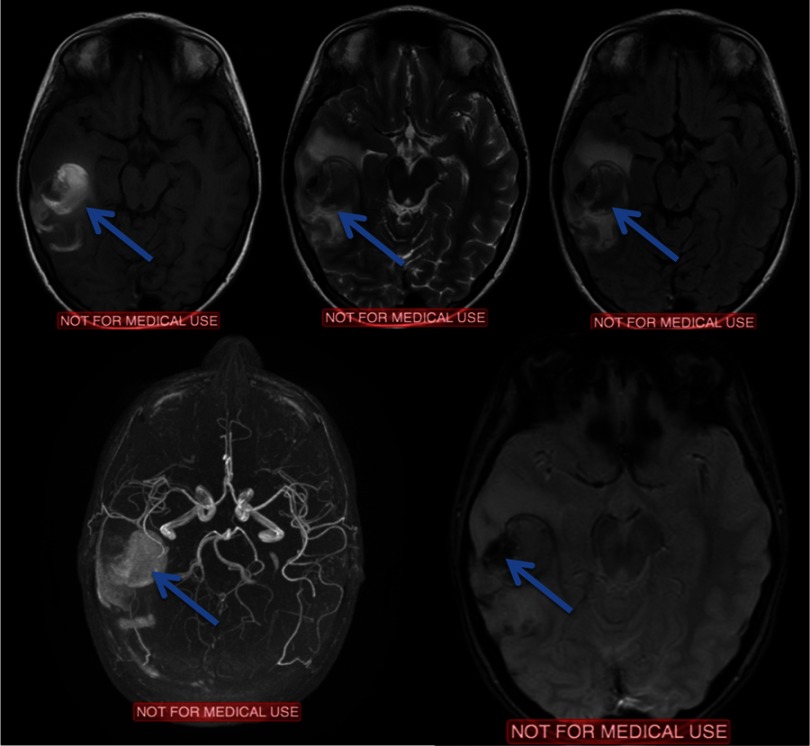
Arrows depict the evolution of signal changes, 10 days after the event. MRI and MRA scan after 10 days. T1W, T2W, FLAIR and T2-GRE images and MRA reconstruction.

An electroencephalography (EEG) study detected focal cerebral disturbances. A repeat MRI scan (with contrast) and MR angiography (MRA), indicated a hemorrhagic infarct in the territory in a subacute phase with related edema (hemorrhagic stroke). MRA further recognized stenosis of the right (middle cerebral artery) MCA with obstruction of the posterior peripheral branches (Figure 3).

**Figure 4.**
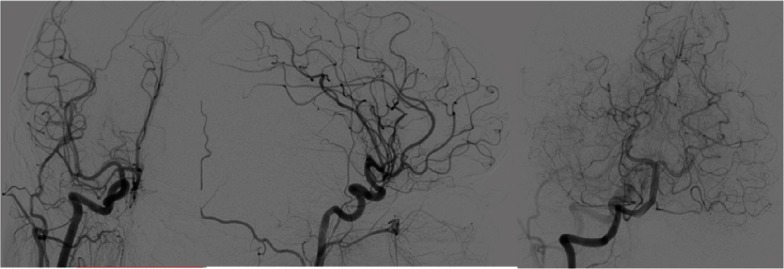
Digital subtraction angiography (DSA) imaging, revealing no pathological findings.

Consequently, a digital subtraction angiography (DSA) from the femoral artery was performed which did not reveal underlying vascular abnormalities (Figure 4).

A thorough investigation for hypercoagulable states (deficiencies of protein C and anti III, protein S, antithrombin and plasminogen, molecular studies for factor V Leiden, prothrombin 20210A, homocysteine, MTHFR gene mutations) and immunological–rheumatological conditions (HLA-51, c-ANCA, p-ANCA, anti-GBM, LA1 and LA2, I*β*-2GPI, ACA IgM and IgG antibodies) did not reveal any abnormalities.

**Figure 5.**
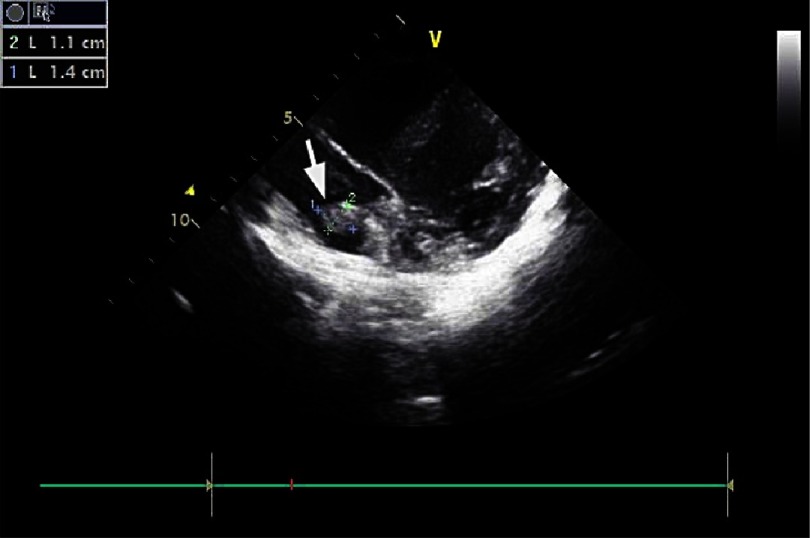
Arrows in Figures 5 and 6 indicate the location of the thrombus with its approximate dimensions.

**Figure 6.**
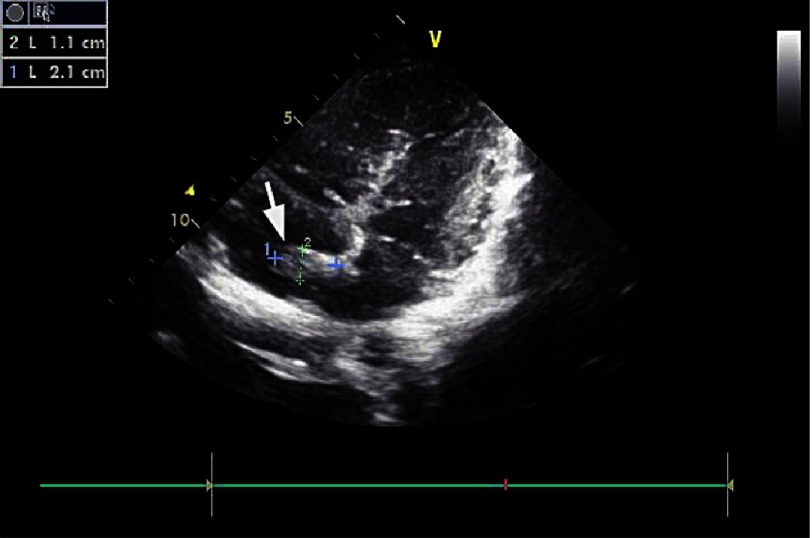
Arrows in Figures 5 and 6 indicate the location of the thrombus with its approximate dimensions.

An ultrasound study of the vessels of the lower extremities revealed an intraluminal thrombus of the left superficial femoral and popliteal vein. A thoroughly detailed investigation of patient’s history revealed a minor sports related blunt injury of the left lower extremity a day before the initial symptoms, which was associated with lower extremities ultrasound findings. Additionally, an echocardiogram visualized a thrombus (2 ×1,5 cm) attached to the right atrium in conjunction with patent foramen ovale (Figures 5 and 6).

**Figure 7.**
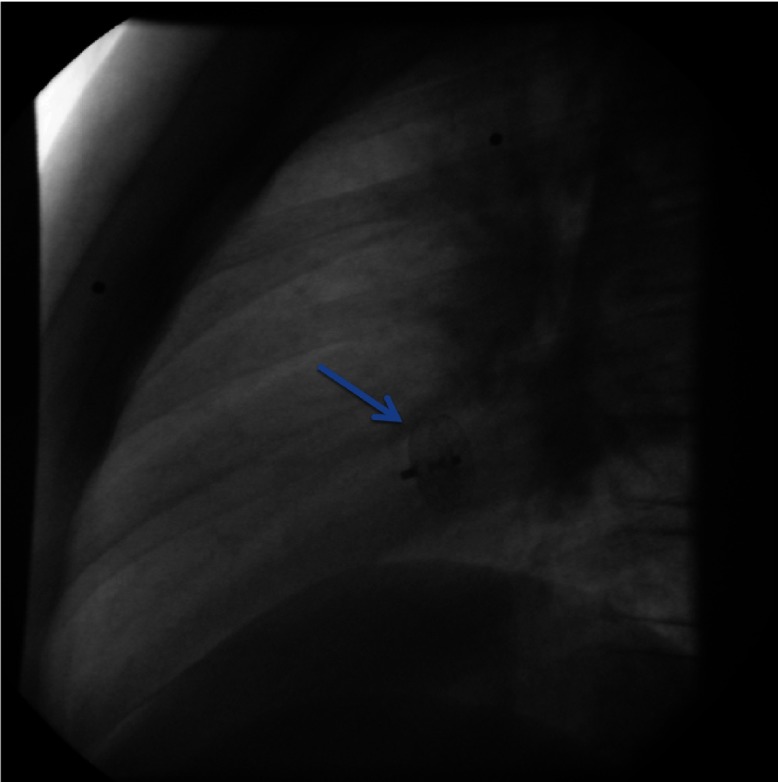
The arrow indicates the site of placement of the umbrella.

Due to the relative contraindication for thrombolysis, patient was initiated immediately on anticoagulation therapy with low molecular weight heparin and warfarin. Two days later, she developed acute symptoms of dyspnea and chest pain and a subsequent spiral thorax CT revealed pulmonary emboli at the left pulmonary artery, as long as the persistence of the atrial thrombus. Anticoagulation therapy was continued and a foramen ovale umbrella placement was later performed (Figure 7).

**Figure 8.**
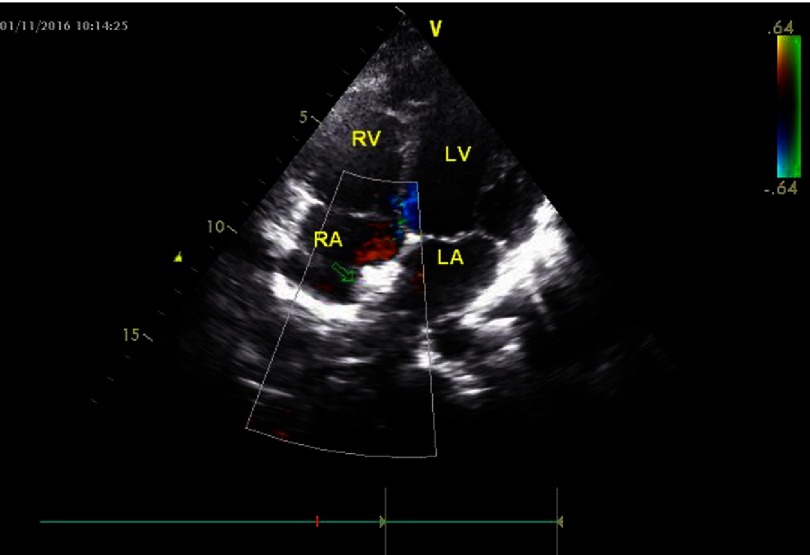
Arrow indicates the position of the umbrella.

Patient remained symptom free in the follow-up period and serial cardiac ultrasound examinations revealed gradual resolution of the right atrial thrombus (Figure 8).

**Figure 9.**
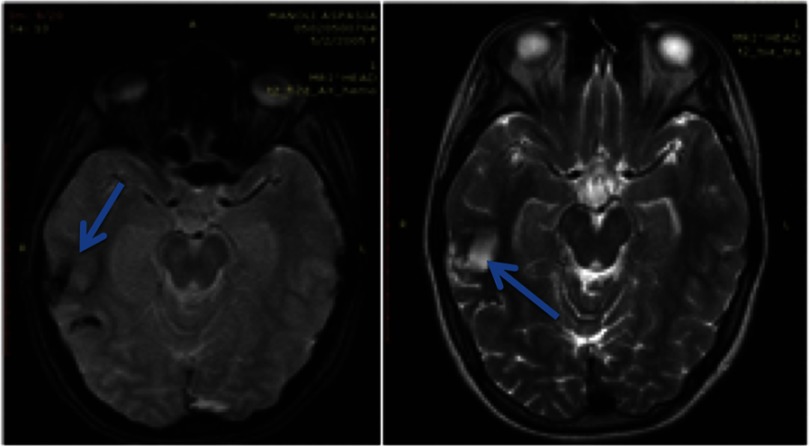
The left image is an MRI GRE sequence, showing the hemosiderin ring (arrow) and the right image is a FLAIR sequence, revealing an area of intermediate to hypointensity signal (arrow).

A few months later, a repeat MRI scan was performed, while the patient being neurologically normal. The examination verified the known lesion at the right temporal-occipital lobe region, which revealed characteristics, compatible with a chronic lesion. More specifically, the imaging of the lesion identified a territory with intermediate to hypo-intensity signal at Flair sequences and hemosiderin ring at GRE sequences (Figure 9).

## Discussion

### Epidemiology—definition of cryptogenic stroke

Arterial ischemic stroke (AIS), with an estimated incidence of 1.1-4.3 per 100,000, is an important cause of morbidity and mortality in children and the recurrence risk is high^1^. It is defined as an acute clinical syndrome with a neurological deficit referable to a cerebral arterial territory and a brain MRI showing a corresponding area of acute infarct.

A stroke is termed “cryptogenic” when its etiology cannot be attributed to any specific cause after an extensive search for the most common causes, such as atherosclerosis of the intracranial vessels, lacunar damage from hypertension, or embolus derived from a thrombus located in the left atrium, the left ventricular apex, or at the level of an ulcerated plaque of the aortic arch.

### Etiology

The etiology of AIS remains undetermined in a high proportion of children. Predisposing conditions for ischemic cerebrovascular accidents in children include congenital heart malformations (congenital cyanotic complex heart malformations or acquired heart disease), sickle cell disease, infections, and collagen tissue abnormalities^2^, but around half occur in children who were previously well (cryptogenic stroke)^3^. It is well known from the literature, that one of the most common conditions associated with AIS is congenital heart malformations (like patent foramen ovale)^4–14^.

### Paradoxic embolism and stroke

Recently, paradoxical embolism across the PFO was suggested as a possible etiology in some of these children^15^. PFO is reported, as an autopsy finding, to remain patent in about 25% of adults, thus presenting a potential passageway for paradoxical embolization. On some particular circumstances such as during Valsalva maneuver, which is reproduced by the act of defecating or coughing, the reversal of the physiologic inter-atrial pressure gradient results in right-to-left shunting across the PFO and contributes to the passage of embolic material. The prevalence of PFO was significantly higher in patients with cryptogenic stroke versus those with known causes of stroke (42% vs 7%), indicating that PFO is associated with cryptogenic stroke^16,17^. The association is documented in case reports^2,3,18–22^. However, the direct role of a PFO in stroke remains unclear^23^.

In our case, the dual (and simultaneous) detection of thrombi in the deep venous system and the right atrium along with the rapid sequence of embolic events in the absence of other underlying pathological conditions, point out the paradoxical embolism through a patent PFO as the most plausible scenario for the ischemic stroke.

### Prothrombotic disorders

Also, prothrombotic disorders are frequently identified in pediatric patients with stroke^7^ and case control studies demonstrate an association of arterial ischemic stroke in children with hereditary prothrombotic risk factors^4^. Another study reports prothrombotic abnormalities to be present in 20–50% of children with arterial ischemic stroke^11,24^.

Reasoning the coexistence of deep venous thrombosis and right atrial cavity thrombus in our patient with the absence of positive laboratory results for hypercoagulable disorders, we speculate either a transient hypercoagulable state, possibly associated with patient’s minor sport related injury or an unidentified mechanism by our thrombophilia screening.

### Deep venous thrombosis and cryptogenic stroke

Young adults with cryptogenic ischemic stroke are more likely to have both patent foramen ovale and pelvic deep vein thrombosis (DVT) than young adults with ischemic stroke of known cause. Young patients with cryptogenic transient ischemic attack (TIA) or stroke and patent foramen ovale (PFO) should be evaluated for lower-extremity or pelvic venous thrombosis, which would be an indication for anticoagulation. In our case, screening for underlying causes of cryptogenic stroke with ultrasound of the lower extremities revealed venous thrombosis. The most probable releasing factor (and causative) of this event was a few days previously reported, sports related, minor lower extremity injury - a finding supported by the literature.

### Right atrial thrombus and stroke

• Right-sided mobile thrombi “in transit” from the deep venous system are found in adult case reports or case series in which clots were detected incidentally or during acute pulmonary thromboembolism.
• A recent pediatric literature review article reports in a sum of 122 cases, 91% of cases to be associated with central venous catheters, 40.8% in premature neonates, 27.2% in post cardiac surgery patients, and 19.2% to have underlying malignancies^25^.
• In our case, the only causative mechanism for the formation of the right-sided thrombus, which was detected upon admission with esophageal ultrasound, was lower extremity deep venous thrombosis, in an otherwise healthy child. A Medline search of Pubmed database using the keywords “right atrial thrombus” and “children or pediatric or paediatric” and “patent foramen ovale” and “stroke” did not reveal any relevant case so, to the best of our knowledge, this is the first documented case of right atrial thrombus resulting from deep venous thrombosis in a pediatric patient with patent foramen ovale and associated ischemic stroke event.

### Treatment guidelines

Young patients with cryptogenic TIA or stroke and PFO should be evaluated for lower-extremity or pelvic venous thrombosis, which would be an indication for anticoagulation. In the setting of a large acute stroke, however, full-dose anticoagulation is not recommended, and an inferior vena cava filter may be the safest alternative. In patients with cryptogenic TIA or stroke, a PFO, and DVT, guidelines from the ACCP currently recommend VKA therapy for 3 months and consideration of PFO closure rather than no VKA therapy or aspirin therapy.

For patients with an ischemic stroke or TIA and both a PFO and a venous source of embolism, anticoagulation is indicated, depending on stroke characteristics (Class I; Level of Evidence A). When anticoagulation is contraindicated, an inferior vena cava filter is reasonable (Class IIa; Level of Evidence C) (New recommendation).

In cases of concomitant venous and arterial embolism that paradoxical embolism is strongly considered, chronic anticoagulant therapy and an inferior vena cava filter can be justified to prevent further recurrences of both pulmonary and paradoxical embolism^26^.

In the incident of right atrial thrombus, different treatment modalities are reported such as surgical thrombectomy, thrombolysis, anticoagulation therapy or observation only, the choice of which depended mainly on underlying etiology^25^.

In our case, because of the hemorrhagic transformation of the cerebral stroke was an absolute contraindication for the initiation of fibrinolytic therapy, anticoagulation with subcutaneous low molecular weight warfarin along with foramen ovale umbrella placement constituted the selected treatment strategy. This strategy proved to be efficacious during the follow-up period.

### Prognosis

Regarding outcome data, it is referred that permanent moderate-to-severe motor or cognitive disabilities occur in 75–87% of children with stroke, and death occurs in 5–28%^14^.

In our case, patient presented with indirect symptoms, such as headache and epileptic fit, these symptoms appearing late from stroke ictus, as seen from the initial MRI presentation of the stroke which was in the hemorrhagic transformation phase and the patient was not on anticoagulation therapy for any reason or didn’t report aspirin uptake. Furthermore, she did not suffer any major clinical and neurologic sequelae from the event. These findings, possibly due to the clinically silent anatomical area of the stroke, are contrary to the majority of the cases described in the literature that have unfavorable neurological prognosis.

## Conclusion

Cryptogenic AIS is a diagnosis of exclusion. The emergence of cases reporting patients with cryptogenic AIS harboring a patent foramen ovale, tends to reveal the presence of an associated causative factor.

For all the aforementioned reasons, and because many of the aspects of the issue of AIS remain unresolved, we consider that it would be meaningful to present a case that mismatches a lot of aspects of the reported clinical cases and promotes a little known pathophysiologic mechanism, supported by a wide range of clinical and laboratory data.
